# An early detection and segmentation of Brain Tumor using Deep Neural Network

**DOI:** 10.1186/s12911-023-02174-8

**Published:** 2023-04-26

**Authors:** Mukul Aggarwal, Amod Kumar Tiwari, M Partha Sarathi, Anchit Bijalwan

**Affiliations:** 1 Dr. A.P.J. Abdul Kalam Technical University, Lucknow, Uttar Pradesh India; 2 Rajkiya Engineering College, Sonbhadra, Uttar Pradesh India; 3 Amity School of Engineering and Technology, Amity University, Noida, Uttar Pradesh India; 4 Faculty of Electrical and Computer Engineering, Arba Minch University, Arba Minch, Ethiopia

**Keywords:** Brain tumor, Segmentation, ResNet, Deep neural network, CNN, Healthcare, Prediction models

## Abstract

**Background:**

Magnetic resonance image (MRI) brain tumor segmentation is crucial and important in the medical field, which can help in diagnosis and prognosis, overall growth predictions, Tumor density measures, and care plans needed for patients. The difficulty in segmenting brain Tumors is primarily because of the wide range of structures, shapes, frequency, position, and visual appeal of Tumors, like intensity, contrast, and visual variation. With recent advancements in Deep Neural Networks (DNN) for image classification tasks, intelligent medical image segmentation is an exciting direction for Brain Tumor research. DNN requires a lot of time & processing capabilities to train because of only some gradient diffusion difficulty and its complication.

**Methods:**

To overcome the gradient issue of DNN, this research work provides an efficient method for brain Tumor segmentation based on the Improved Residual Network (ResNet). Existing ResNet can be improved by maintaining the details of all the available connection links or by improving projection shortcuts. These details are fed to later phases, due to which improved ResNet achieves higher precision and can speed up the learning process.

**Results:**

The proposed improved Resnet address all three main components of existing ResNet: the flow of information through the network layers, the residual building block, and the projection shortcut. This approach minimizes computational costs and speeds up the process.

**Conclusion:**

An experimental analysis of the BRATS 2020 MRI sample data reveals that the proposed methodology achieves competitive performance over the traditional methods like CNN and Fully Convolution Neural Network (FCN) in more than 10% improved accuracy, recall, and f-measure.

## Introduction

Brain Tumor segmentation and detection are very challenging in the medical imaging area. Various DNN methods are used for Tumor segmentation, utilizing multiple deep-learning network architectures. The processing of medical images plays a crucial role in assisting humans in identifying different diseases [1]. Classification of brain Tumors is a significant part that depends on the expertise and knowledge of the physician. An intelligent system for detecting and classifying brain Tumors is essential to help physicians. Gliomas have an irregular shape and ambiguous boundaries, which are the most challenging Tumors to detect. Various authors have performed additional research on deep learning networks based on healthcare, i.e., Convolutional neural networks (CNNs), LinkNet, Visual Graphic Group (VGG), UNet, and SegNet [2].

Image segmentation poses significant challenges, including categorization, image processing, object recognition, and explanation. Whenever an image classification model is formed, e.g., it must be eligible to function with great precision even when subjected to occlusion, lighting modifications, observing angles, and other factors [3].

The conventional object detection process, including its primary feature extraction step, is unsuitable for wealthy areas. Sometimes experts in the domain cannot provide a single or collective of functionalities capable of achieving accurate results under varying conditions. The concept of model training emerges due to that kind of problem. The appropriate features for working with image data are instantly figured out [4].

Content-based image retrieval provides various imaging modalities, such as CT, MR, PET, X-rays, and Ultrasound. Also, the many image data available because of different scan parameter settings and multiple views of the same pathology make image retrieval in the medical domain tough and challenging. However, at the same time, it is one of the essential applications [5]. The MR images are taken from three different directions. These views are called sagittal, axial, and coronal [6]. For CBIR to be used in healthcare as a diagnostic aid, the medical information framework must be robust in various scenarios to be accepted by clinicians and medical practitioners [7].

First, case-based reasoning will be more acceptable to the medical community when the retrieval engine results in cases with exact locations and similar pathology responding to a query (new) case [8].

This will significantly help the medical expert have more information about the case and aid the expert in monitoring. Secondly, the database formed for testing purposes should be carefully built consisting of cases from multiple views, different scanning parameters, and acquired from different imaging modalities. CNN has been used to segment Tumors in multi-modal Imaging [8].

The CNN architecture is sophisticated, combining segmentation and classification into a single product. Current segmentation methods have been designed to solve the reduplication issue of CNNs by allocating a target class toward each pixel. A CNN model has been transformed into an FCN (Fully CNN). This article has critical contributions to brain Tumor research, which are as follows:

- This research develops the ResNet Model to address the weaknesses of CNN and FCN methodologies and improve computational costs. The principle of ResNet is premised on adding the layer’s outcome towards its significant input.
- The simple transformation used in Enhanced ResNet mainly improves the training process of Convolutional models by utilizing the “shortcut links.” These links provide all the possible route details in a single place and provide access in a single click reducing the accessing time.

The complete research article is organized as follows: Section 1 covers the introduction, Section 2 covers existing Tumor segmentation work related to research, Section 3 covers material and methods, section 4 covers results, section 5 covers the discussion and Section 6 covers the conclusion and future direction of the research.

## Related works

The field of Tumor segmentation is continuously undergoing investigation. Deep learning has recently proven effective in healthcare image segmentation and information extraction. In deep learning techniques, pixel-based classification is the latest phenomenon. Various researchers have suggested different methods for brain Tumor segmentation. This section covers the analysis of a few of the critical research.

Research [9] presents brain Tumor segmentation using DNN. Brain Tumors are segmented on magnetic resonance visuals of the brain using a Deep Convolutional encoder model. This approach enhances learning by extracting attributes from complete images, eliminating patchwork selections, and improving calculations at adjacent intersections. Research [10] presented a technique for the early detection of brain cancers. Magnetic resonance images were examined to identify Tumor-bearing areas and categorize them into various classifications. In image classification techniques, deep learning generates efficient performance.

Consequently, the Fully Convolutional Networks technique was applied and incorporated through the Tensor Flow repository throughout this research. A newer CNN technique has been demonstrated to have a precision of 91 percent, which is better than previous research.

Research [11] developed a model by utilizing Brain imaging to recognize the nature of brain Tumors. A two-dimensional CNN was used to acknowledge malignant Tumors with an accuracy rate of 93 percent. The data for the four most often detected brain Tumors are included in the research’s analysis.

Research [12] advised a responsive and efficient Tumor segmentation framework. In a Cascades Classification Model, this strategy reduces computation time and addresses the problem of overfitting. Using two separate forms, this CNN architecture extracts global and regional characteristics. Additionally, the Tumor detection precision is significantly enhanced compared to current algorithms. The average WT, increasing Tumor, and Tumor center dice scores for the proposed approach achieved 92.3%, 94.5%, and 93.2 %.

Research [13] developed a model to evaluate Tumors utilizing an MRI dataset. It entails finding cancer, grading it by size and type, and determining the Tumor’s position. Instead of using alternative approaches for each classification task, this strategy used a single model to organize MRI Images on many classification techniques.

Research [14] prompted brain Tumor identification and separation by integrating both training methods. The first proposed approach was the Binary Pattern method based upon that neighbor range connection termed ‘nLBP’. The second strategy was based on the perspective of the neighbor next door called “αLBP.” The above two techniques were developed to process and analyses MRI images of the most prevalent cancers: Glioblastoma, malignant Tumors, & gland Tumors. For feature evolution, the statistics of the precompiled images were employed. Conventional extraction of feature strategies scored worse than this proposed model.

Research [15] applied the brain Tumor partition by integrating all the RELM (“Regularized Extreme Learning Machine”). The procedure initially normalized images to make the framework’s understanding easier. The framework utilized a min-max strategy for pre-processing phase. This min-max processing method significantly improved the brightness of the original images.

Research [16] applied the brain Tumor partition by integrating all the RELM (“Regularized Extreme Learning Machine”). The procedure initially normalized images to make the framework’s understanding easier. The framework utilized a min-max strategy for pre-processing phase. This min-max processing method significantly improved the brightness of the original images.

Research [17] proposed a Convolutional Perceptron neural network-based segmentation initiative to improve the Whale Optimization method. For improved feature evolution and partition, the hybrid algorithm produced an updated form of WOA. The Mean Filtering was used to first remove the noise from data in product development and production. The enhanced WOA was used to pick characteristics from the retrieved features. The MLP-IWOA-based classification was used to classify Tumors and outperformed specific current approaches.

Research [18] consolidated significant statistical attributes with CNN architectures to create a technique for the segment of brain cancer cells. The architecture concentrated on the Tumor’s boundary. The two-dimensional Wavelet Decomposition, Gabor Filters Filter, and similarity measures were used to identify and extract the image. A significant feature with further categorization was developed by combining these statistical properties.

Research [19] analyzed that cancer seems to be the most severe disease and therefore is considered challenging to treat. While behind the bottom section of the belly is a pancreatic malignant that develops in the pancreatic cells that aid indigestion. Its stage of growth determines the therapy for this Tumor. The Tumor is detected by individually identifying the afflicted region of the CT scanned data. It forecasts the Tumor region under consideration by utilizing Gaussian Mixture Framework and Expectation-Maximization method & CNN [20].

### Materials & Methods

This section covers the essential methods used in this research and the proposed improved ResNet method working.

### Convolution Neural Network

CNN is mainly a deep learning approach used to classify images. CNN is an artificial neural network designed to analyze input in a mesh form. In CNN, a Convolution process is an activity inside the convolution layer premised on just a mathematical matrix operation that increases the matrix of both the filtration system in the image to be analyzed. This convolution operation is the first and most significant utilization phase [21].

**Fig. 1:**
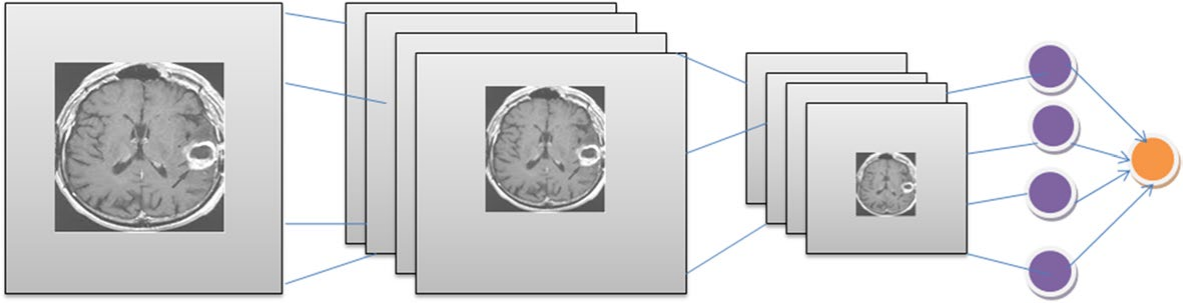
Architecture of Convolution Neural Network (CNN)

Figure 1 shows the architecture of CNN. This figure shows three layers named convolutional, pooling and fully connected layers. Another layer often employed is a pooling layer that receives the whole or averaged values of the pixels image regions. CNN is capable of learning advanced functionality by creating a feature map.

It constructs many feature maps; each convolution layer core is covered across its input sequence. Input sequences recognize characteristics presented on this feature map as simple boxes. Such maps are sent to the optimum related resources layer, keeping the most important features while discarding the remaining. Inside each fully-connected layer, the characteristics of its max-pooling base layer are turned into a 1-D feature vector, which will be employed to determine the output consequence [22]. Image scalability is not possible in a traditional neural network model.

However, in a CNN model, the image can be scaled (that is, it can go from a 3D input space to a 3-dimensional output pattern). The CNN Model comprises its input layers, convolution, Rectified Unit layer, pooling layer, and fully-Connected layers. The provided data (input images) gets split into small sections inside the convolution operation. The ReLU layer performs element-by-element activation. The requirement for a pooling layer is voluntary. Here the option of using or skipping can be taken

On the other hand, this pooling layer is mainly utilized for downstream sampling. A category score or class score code is represented in the last stage (i.e., fully connected layer) based on 0 and 1. The CNN-based brain Tumor segmentation training/testing rounds are categorized into two sections. All images are classified using categories like Tumor images and non-Tumor brain Tumor images [23].

Algorithm: 1 CNN-based Brain Tumor segmentation process. Input: Brain Tumor imagoes dataset Output: Tumor images are segmented into Tumor and Non-Tumor images. Step 1: Impose a Convolutional filtration to the very initial layer. Step 2: Refine the Convolutional filter to lower its sensitivities called “sub-sampling.” Step 3: All signal transmissions from one layer to the next are regulated primarily through activation blocks. Step 4: Use the rectified linear component to shorten the training process. Step 5: Each neuron in the previous layer is linked to every cell inside the subsequent stage. Step 6: At the end of the learning process, a failure layer is applied to provide constructive feedback on the CNN architecture.

### Fully Convolutional Network (FCN)

In research [24], the FCN has been suggested as a solution to semantic segmentation and classification. Researchers utilized AlexNet, VGGNet, and GoogleNet as potential options. Researchers transmitted all such approaches from classification methods to thick FCN by replacing convolution layers with (1×1) Convolutional layers and adding a (1 × 1) convolution to frequency axis 21 to forecast rankings at each class and context category. FCN can learn to quickly build dense assumptions for per-pixel processes such as semantic segmentation [24].

**Fig. 2:**
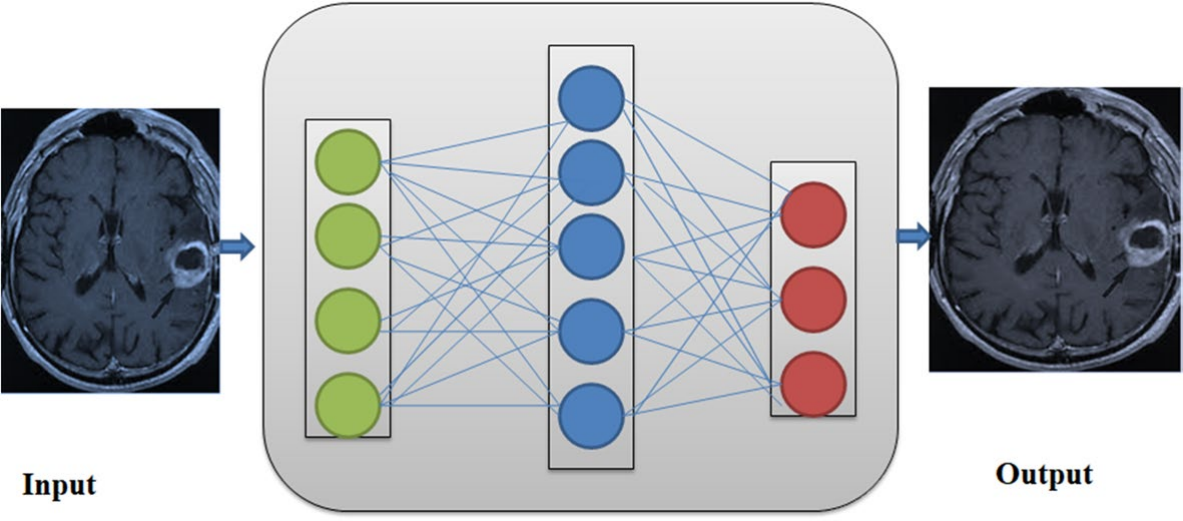
FCN Architecture

Figure 2 shows the working of FCN architecture for image segmentation. Each layer in FCN is just a 3-D array of different sizes, including height, width, and dimension. The image is the first layer, with all the pixels’ information, including height, width, and colour space dimensions. Higher-level locations correlate to the image regions and are route-based, their visual field.

Significant alterations in FCN that further contributed to the conceptual framework to accomplish state- of-art outcomes are just the prototype VGG16, bipolar extrapolation method for up-sampling only the resulting feature outline, and skip correlation for incorporating minimal layer as well as consistently high layer characteristics in the closing layer for fine-grained segmentation. FCN only uses local data for segmentation.

However, only neighborhood details make logical segmentation unclear because the image’s global semantic scope is lost. Relevant information first from the entire image is beneficial for reducing uncertainty. U-Net and V-Net are the most popular FCN architectures widely used in image segmentation [25, 26].

### Proposed model based on Residual Learning Network

The work explains the MRI brain Tumor datasets for medical image analysis that are freely available. This research outlines the performance indicators for evaluating deep learning image and segmentation models.

To address existing challenges, this work utilized an advanced pre-processing approach in the proposed method to eliminate many irrelevant data, resulting in impressive outcomes, perhaps in the current convolutional neural network.

The proposed strategy does not employ a complicated segmentation method to categorize the position of the brain Tumor and the extraction of features, which results in a time-consuming process with a high fault rate.

**Fig. 3:**
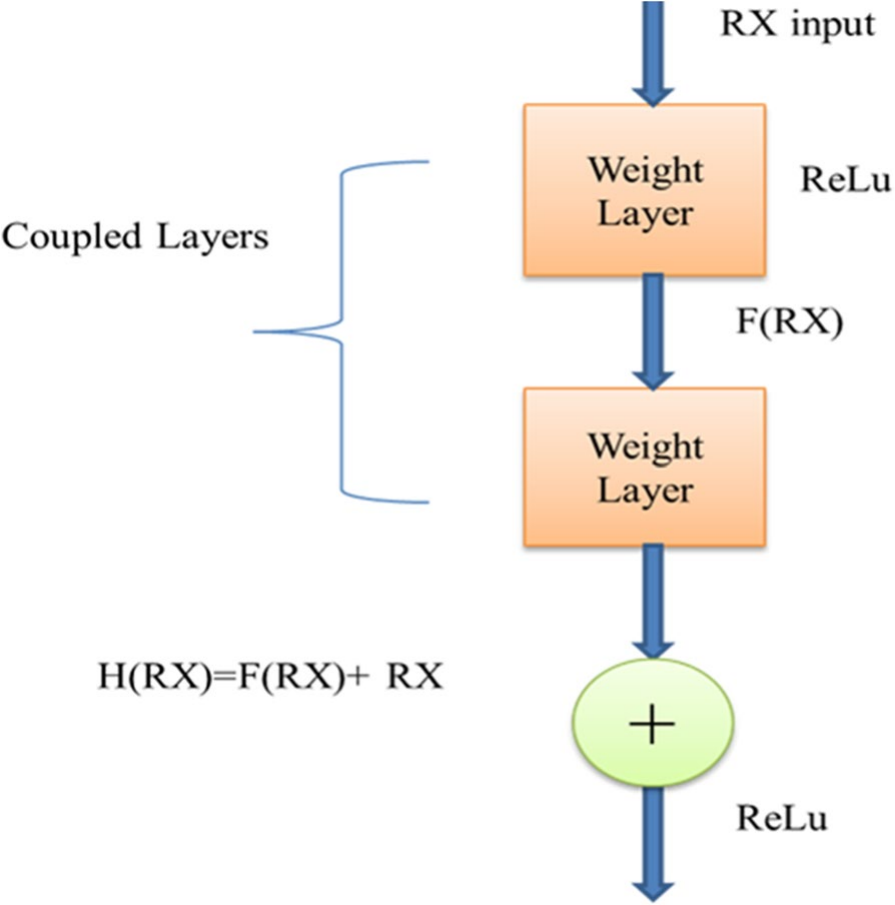
ResNet working structure

ResNet has been taken for proposed work as it is free from gradient issues, originally a problem of various deep learning models. The fading gradient problem occurs during the training procedure of a CNN network. As the learning continued, a gradient rule of previous layers lowered to nil or zero. A ResNet method can be utilized to address this problem. A gain of the relationship between these factors residual layer in ResNet is combined with all of its direct input to become its next inner layer [27–29]. Let H(RX) denote a residual mapping to establish a deep residual block, as shown in Fig. 3.

1 $$\mathrm{H}(\mathrm{RX})=\mathrm{F}(\mathrm{RX})+\mathrm{RX}$$

Consider a CNNS block with RX as input and the main objective of learning the accurate distribution H (RX). The output and the information difference is the “Residual learning value (RL),” as described in equation 2.

2 $$\mathrm{RL}(\mathrm{RX})=\mathrm{H}(\mathrm{RX})-\mathrm{RX}$$

where H (RX) represents the actual outcome, RL represents the Residual learning value, and RX represents the input. To overcome the gradient issue of DNN, this research provides an efficient method for a brain Tumor.

### The Proposed Improved ResNet Model Working

Segmentation based on the Improved Residual Learning Network (ResNet). Existing ResNet can be improved by maintaining the details of all the available connection links. The proposed ResNet utilizes a jump relationship in that initial input data is combined with the convolution building’s outcome. The above addresses the disappearing gradient problem by enabling an additional route for the gradient to move across. The proposed method also utilizes an identification function that allows a more significant layer to accomplish as delicate as a bottom level. The proposed model used the pre-processing, Data Segmentation, and post-processing phases [30–32].

**Fig. 4:**
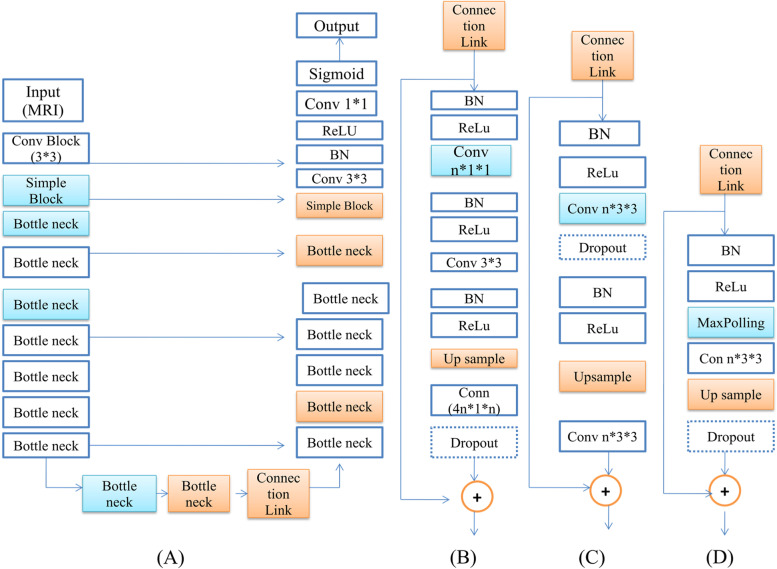
(**A**) Long Skip Connection process in ResNet, (**B**) ResNet Bottleneck Block process, (**C**) ResNet Basic Block Working, and (**D**) ResNet Simple Block Working

Figure 4 presents the working of the proposed ResNet model. In improved ResNet, the complete process is divided into four phases

In past research, researchers suggested numerous ResNet configurations with ResNet-18, ResNet-34, ResNet-50, and ResNet-152 layers. Each layer of just a ResNet consists of several frames or building blocks. The Identification and Convolutional blocks are merged to produce an Improved ResNet structure in such implementations. This research uses an improved ResNet-50 layered model for segmentation because it has more fabulous depth layers than ResNet-34 and fewer parameters than other ResNet models, resulting in a quicker training period. Figure 4 shows the ResNet-50 architectures [33].

3 $${L}_{bce}=\sum_{i}^{0} yi*logOi+\left(1-yi\right)*\mathrm{log}\left(1-Oi\right)$$

4 $${L}_{dice}=-\frac{2\sum_{i}^{0}*(Oi*yi) }{\sum_{i}^{0}Oi+\sum_{i}^{0} yi}$$

where ${\mathrm{L}}_{\mathrm{bce}}$ represents the standard binary entropy loss and ${L}_{dice}$ represents the dice loss mainly occurring during image segmentation.

The complete process of the proposed Improved ResNet is as follows:

- Step 1: It contains a two-dimensional Convolution that has 64 filtrations of (7*7) framings and just a stride of size (2*2) small-batch Standard, and also the ReLU (activation function) completes the route axis uniformity. Finally, a Max Pooling with a frame of (2*2) is used.
- Step 2: It includes one two-dimensional CNN model block with two Identification blocks, each having three pairs of filtrations [64, 64, 256] and a stride with size (1*1).
- Step 3: It comprises one fully-connected block with three Identification blocks, each with three pairs of filtrations [128, 128, 512] to a stride with size (2*2).
- Step 4: It contains one Convolution layer block as well as five Identification; it also uses three pairs of filtration of size [256, 256, 1024] and blocks size (3*3), as well as a stride of size (2*2).
- Step 5: It comprises one Convolution layer block and two Identification blocks, each with three pairs of filtrations [512, 512, 2048] with just a stride size (2*2).
- Step 6: The fully connected layer is also used to reduce the direct input toward the number of subclasses using a “Soft-max reactivation” algorithm, after which the outcome is flattened.

### Proposed work model description

#### Phase 1

The Residual Network with Long Skip Connections is represented by Phase 1. It contains down-sampling (in Figure 4, represented by blue colour), indicating that it is a contracting path. Similarly, an up-sampling (in Figure 4, represented by orange colour) reveals that it is a rapidly expanding route. During this process, long skip connections interact with the contracting path to the growing direction, shown with arrows from left to right in Figure 4A.

#### Phase 2

Various (1*1) and (3*3) Conv are used; these blocks are called bottlenecks. BN and ReLU are used in this phase [34–36]. The concept behind Pre-Activation ResNet is to employ BN-ReLU just before a Conv, as shown in Figure 4B. the Benefits of using these bottleneck blocks are less training time and improved performance. The use of a bottleneck reduces the number of parameters and matrix multiplications. For example, if 9 operations were there, it would mainly reduce them to 6. The idea is to make residual blocks as thin as possible to increase the depth and has fewer parameters.

#### Phase 3

The third phase is the primary block phase, mainly utilizing (3*3) blocks only, not the (1*1) block. This phase represents the basic block. A basic ResNet block comprises two layers of 3x3 conv /BatchNorm/relu. In the picture, the lines represent the residual operation. The dotted line means that the shortcut was applied to match the input and the output dimension

#### Phase 4

The last phase is the simple block phase, which utilizes (3*3) n blocks. Max Pooling is used in this phase which rejects a big chunk of data. It extracts only the most salient features of the data. MaxPool bound the system to only the very important features and might miss out on some details

### Dataset description

**Fig. 5:**
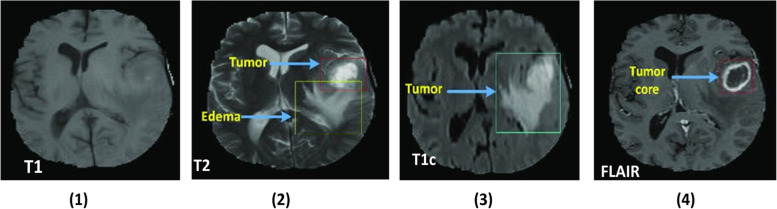
Brain Tumor Images in BraTS2020 (1) for Type T1, (2) for Tumor Type T2, (3) for Tumor Type T1c, and (4) for Tumor type FLAIR

This research utilized the BraTS2020 dataset [37]. A brat consistently evaluates cutting-edge brain Tumor segmentation approaches in composite MRI scan data. BraTS 2020 uses multi-institutional like pre Image data. It concentrates on segmenting inherently heterogeneous (through shape, location, and cell biology) brain Tumors, such as gliomas. It includes 369 brain Tumor MR images. As described in Fig. 5, all previous research examined T1-weighted (called T1), post-contrast T1-weighted (called T1ce), T2-weighted (called T2), and fluid-attenuated inversion recovery (called Flair) sequencing. Each of the images has a (240*240*155) size[38]. The dataset is collected from the online Kaggle website. It includes 369 brain MR images; 125 are utilized for training and 169 MRI images for testing. Figure 5 shows the Brain Tumor types available in the BraTS 2020 dataset.

### Performance measuring parameters

The following essential version was utilized to measure the performance of the proposed method and the existing one [39–41].

#### Mean Square Error (MSE)

The procedure of squaring predicted quantities is MSE. An average of such squared errors can be used to explain it. Equation 5 denotes the cumulative square estimation error between the actual picture and the output image as MSE

5 $$MSE=\frac{1}{MN} *\{\sum_{i=0}^{m-1}*\sum_{j=0}^{n-1}[l\left(i,j\right)-K\left(i,j\right)]{\}}^{2}$$

#### Peak Signal Noise Ratio (PSNR)

PSNR relates to a picture’s immune function to noise external interference signals. When the PSNR level is greater, the noisy interference signal’s effect on the MR image database is minimal. MSE phrases are used to represent PSNR. PSNR must be between 40 and 60 dB. It is calculated by Eq. 6. Where Maxl is usually 255 and MSE is the mean square error

6 $$PSNR=10log10\frac{Max1}{MSE}$$

#### Computation Time

The time it takes to complete the segmentation procedure is calculated in milliseconds or Seconds and represented as elapsed time.

#### Jaccard Coefficient (JC)

It also serves as a metric for evaluating segmentation strategies. Jacquard offers Eq. 7 to compute the matching of two Q1 and Q2 pairs by standardizing the volume of their overlap over the respective union.

7 $$JC=2*\frac{|Q1 \bigcap Q2|}{\left|Q1\right|+\left| Q2\right|}$$

#### Dice Similarity Coefficient (DSC)

The DSC is now the most popular and common assessment indicator for assessing the segmentation results and their base facts. This measures the overlap values of two pairs, Q1 and Q2, via normalizing them well across the average of respective standard sizes. DSC is presented in the equation

8 $$Specificity =\frac{TN}{ TN+FP}$$

#### Sensitivity and Specificity

The following Eqs. 9 and 10 calculate sensitivity and specificity as rule-based decision theory measures. Where: TP-True Positive, FP-False Positive, TN-True Negative, FN -False Negative

9 $$Sensitivity=\frac{TP}{TP+FN}$$

10 $$Specificity =\frac{TN}{ TN+FP}$$

## Results

### Training results

In this research, the BraTS2020 dataset has been used collected from Kaggle [35]. This dataset mainly contains 369 brain Tumor patient MR images, where 125 are utilized for training and 169 MRI images for testing. The proposed improved ResNet model, existing CNN model, and FCN (model type U Net) are implemented using Python programming (Tensor flow) in the Anaconda environment. A complete experimental process is divided into two phases: training and testing. The first training phase is applied to train the model.

In the first phase, the normalization process is used. The dataset was corrected in the initial stage because the dataset had some inclination sub-field contortion for which the N4ITK technique has been taken. This technique mainly converts all four MRI brain Tumor image sequences of a particular patient, which helps in Tumor growth and sequencing analysis.

**Fig. 6:**
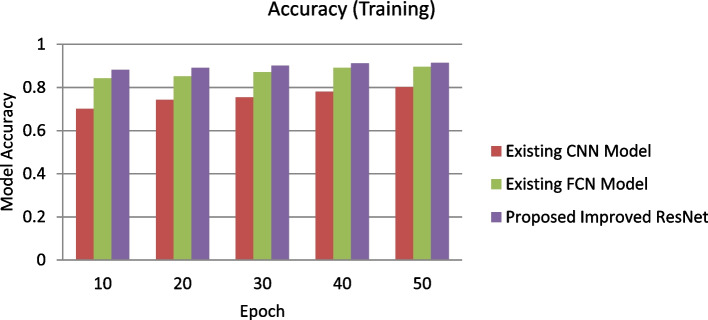
Experimental outcomes for training accuracy of proposed improved ResNet and existing CNN and FCN

This work has presented an improved Recurrent neural network-based approach for Tumor segmentation from multi-modal 3-dimensional MRI images that further utilizes the BraTS 2020 brain Tumor dataset for performance validation. Several possible solutions have been tried while messing with CNN models. Table 1 shows the proposed improved ResNet system parameters utilized for training purposes. After normalization, the Stochastic Gradient Descent optimization method (SGDOM) manages the loss function limit. Its value mainly depends on the gradient (negative) towards the model minima. The training performance of the proposed improved ResNet and existing CNN and FCN is described in Figure 6.

**Table 1:** Training parameters of the proposed improved ResNet model

Phase /steps	Hyperparameter	Parameters value
Initialisation step	Bias	0.1
	Weights	Xavier
ReLU	(α)	0.333
Drop out block	LGG	0.111
	HGG	0.555
Training step	Number of Epochs for LGG and HGG	50
	Batch size	128
	Initial € value	0.004
	Final € value	0.00004
Post Processing stage	Batch Size	128
	Tvol-HGG value	10,000
	Tvol-HGG value	3,000

The proposed enhanced ResNet model shows a lower error rate and higher accuracy in the training phase than existing methods. The proposed improved ResNet model is validated using thirty percent of the training dataset in this experiment.

### Testing results

**Fig. 7:**
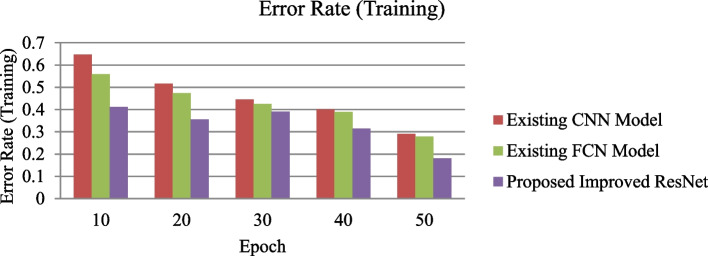
Experimental outcomes for training Error Rate of proposed improved ResNet and existing CNN and FCN

**Fig. 8:**
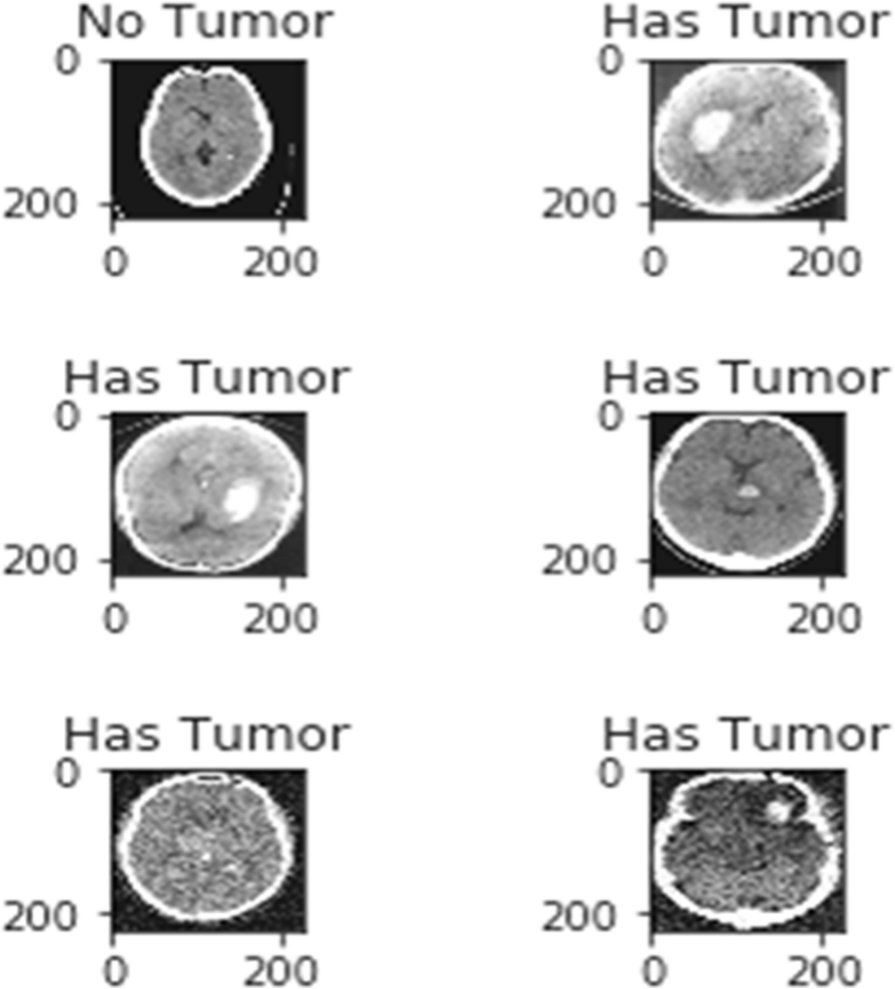
Experimental Results of proposed Improved ResNet Mode

Figure 7 represents the performance validation of the proposed improved ResNet model with 50 epochs. Experimental outcomes prove that the training error rate decreases linearly, and the accuracy percentage increases for each epoch. The test dataset is implemented to the proposed and existing model through the testing phase to identify the brain Tumor cells in MRI images. The proposed improved ResNet model is compared to specific other existing methods in terms of performance metrics (T, ET, WT) to analyze the performance of Tumor segmentation. All performance measures have been taken for each patient in the given dataset. The mean values of these performance measures were then calculated for all patients. Figure 8 shows the experimental results of the proposed Improved ResNet Mode.

## Discussions

Brain Tumor segmentation and detection is a widely known area of research. Various Deep learning models have been executed for all brain Tumor cases like core Tumor region(CT), enhanced Tumor region(ET) and whole Tumor region(WT).

The proposed Improved ResNet model is based on Linked, which further performs identity mapping, and one “s outcome is merged with the outcome of the convolution layer without using any model factors. It also implies that a layer in the ResNet prototype tries to understand the residual of interconnects.

In contrast, layers in CNNs and perhaps FCN (U-Net) methods discover the actual performance. Consequently, the gradients can move quickly back, leading to faster computation than CNNs and FCN models. The quick access links in the proposed Improved ResNet model regulate the disappearing gradient issue.

Tables 2, 3, and 4 compare proposed ResNet and existing models (CNN and FCN) for JC, DICE Score, and Sensitivity, Specificity, and Accuracy parameters for CT, ET and WT respectively on BraTS2020 datasets.

**Table 2:** Comparison of Existing and proposed improved ResNet model for Core Tumor Region (CT)

Core Tumor Region (CT)			
**Performance Measuring Parameter**	**Existing CNN Model**	**Existing FCN Model**	**Proposed Improved ResNet**
JC	0.6485	0.6225	0.658
DICE Score	0.9245	0.889	0.924
Sensitivity	0.7815	0.7256	0.7613
Specificity	0.831	0.814	0.835
Accuracy	0.814	0.789	0.854

**Table 3:** Comparison of Existing and proposed improved ResNet model for Enhanced Tumor Region (ET)

	**Enhanced Tumor Region (ET)**		
**Performance Measuring Parameter**	**Existing CNN Model**	**Existing FCN Model**	**Proposed Improved ResNet**
JC	0.6515	0.6645	0.6328
DICE Score	0.941	0.895	0.945
Sensitivity	0.7989	0.74589	0.7989
Specificity	0.854	0.865	0.926
Accuracy	0.854	0.814	0.913

**Table 4:** Comparison of Existing and proposed improved ResNet model for Whole Tumor Region (WT)

	**Whole Tumor Region (WT)**		
**Performance Measuring Parameter**	**Existing CNN Model**	**Existing FCN Model**	**Proposed Improved ResNet**
JC	0.6695	0.6785	0.6308
DICE Score	0.879	0.874	0.864
Sensitivity	0.7648	0.7465	0.7365
Specificity	0.854	0.846	0.923
Accuracy	0.825	0.826	0.879

According to the assessment conducted for CT proposed model, the output is 0.658, 0.924, 0.7613, 0.835, and 0.854 of JC, DICE Score, Sensitivity, Specificity and Accuracy, respectively. Similarly, the ET proposed model is 0.6328, 0.945, 0.7989, 0.926, 0.913, and for WT, it gives 0.6308, 0.864, 0.7365, 0.923, 0.879 values.

These results show improvement over CNN and FCN due to the four-phase process of the proposed model. The proposed Improved ResNet Model has better outcomes for all three Tumor cases (ET, CT, and WT). This proves that the proposed Improved ResNet model performs well in pediatric segmentation for a brain Tumor. Table 5 demonstrates that the proposed Improved ResNet model has the lowest computation time and the best PSNR and MSE. The proposed method has better results for MSE and PSNR than existing CNN and FCN methods. Loewe, the MSE value shows better performance. The proposed method has 26. 898% MSE and 21.457% PSNR are more than 20%, far better than CNN and FCN.

**Table 5:** Experimental results of Existing and proposed improved ResNet model for Enhanced Tumor Region (ET)

Performance Measuring Parameter	Existing CNN Model	Existing FCN Model	Proposed Improved ResNet
MSE	28.647	33.9478	26.898
PSNR	30.789	29.898	21.457
Computation Time (in Minutes)	112	214	74

## Conclusion & future work

Deep Neural Networks (DNNs) are very useful for image segmentation. However, this technique encounters a disappearing gradient issue that emerges throughout the training. To address this issue, the Improved ResNet is proposed in this research. A “connection link” inside a current ResNet allows the gradient to propagate backwards to subsequent layers. These links provide all the possible route details in a single place and provide access in a single click reducing the accessing time. This paper presents a pre-processing approach in the proposed method to eliminate many irrelevant data, resulting in impressive outcomes.

The proposed Improved ResNet and existing CNN and FCN models are implemented using tensor flow and tested on the BraTS2020 dataset. Experimental results demonstrate the strength of the proposed method in terms of better accuracy, less computation time, MSE, PSNR, and better DSC and JC. The strength of the proposed improved ResNet model is that users did not require the assistance of an expert to manually find the Tumor pixel by pixel, which is a complex and time-consuming operation. This proposed model tackles these issues by utilizing shortcut connection links in ResNet.

The experimental outcomes achieve better performance and a remarkable result compared with conventional techniques. In the binary classification problem, accuracy and precision were examined, as was the Dice coefficient score throughout the segmentation experiment. Future research can improve current outcomes and leverage deeper architectures to improve the overall effectiveness of segmentation output.

## Data Availability

This work utilizes the online brain Tumor available dataset data from the Kaggle BraTS2020 competition. The following is the link: https://www.kaggle.com/datasets/awsaf49/brats20-dataset-training-validation (accessed on 13 March 2022).

## Abbreviations

MRI: Magnetic resonance image
DNN: Deep Neural Networks
ResNet: Residual Network
FCN: Fully Convolution Neural Network
VGG: Visual Graphic group
RL: Residual learning value
CT: Core Tumor Region
MSE: Mean Square Error
JC: Jaccard Coefficient
MR: Magnetic Resonance
PET: Positron emission tomography
TP: True Positive
FP: False Positive
TN: True Negative
FN: False Negative
WT: Whole Tumor Region
ET: Enhanced Tumor Region
PSNR: Peak Signal Noise Ratio
DSC: Dice Similarity Coefficient
SGDOM: Stochastic Gradient Descent optimization method
RELM: Regularized Extreme Learning Machine
